# Fitness Movement Types and Completeness Detection Using a Transfer-Learning-Based Deep Neural Network

**DOI:** 10.3390/s22155700

**Published:** 2022-07-29

**Authors:** Kuan-Yu Chen, Jungpil Shin, Md. Al Mehedi Hasan, Jiun-Jian Liaw, Okuyama Yuichi, Yoichi Tomioka

**Affiliations:** 1 School of Computer Science and Engineering, The University of Aizu Fukushima, Aizuwakamatsu 9658580, Japan; m5252102@u-aizu.ac.jp (K.-Y.C.); mehedi_ru@yahoo.com (M.A.M.H.); okuyama@u-aizu.ac.jp (O.Y.); ytomioka@u-aizu.ac.jp (Y.T.); 2 Department of Information and Communication Engineering, Chaoyang University of Technology Taichung, Taichung 41349, Taiwan; jjliaw@cyut.edu.tw

**Keywords:** deep transfer learning, Yolov4, Mediapipe, machine learning, fitness detection, pose detection, image processing, deep neural network

## Abstract

Fitness is important in people’s lives. Good fitness habits can improve cardiopulmonary capacity, increase concentration, prevent obesity, and effectively reduce the risk of death. Home fitness does not require large equipment but uses dumbbells, yoga mats, and horizontal bars to complete fitness exercises and can effectively avoid contact with people, so it is deeply loved by people. People who work out at home use social media to obtain fitness knowledge, but learning ability is limited. Incomplete fitness is likely to lead to injury, and a cheap, timely, and accurate fitness detection system can reduce the risk of fitness injuries and can effectively improve people’s fitness awareness. In the past, many studies have engaged in the detection of fitness movements, among which the detection of fitness movements based on wearable devices, body nodes, and image deep learning has achieved better performance. However, a wearable device cannot detect a variety of fitness movements, may hinder the exercise of the fitness user, and has a high cost. Both body-node-based and image-deep-learning-based methods have lower costs, but each has some drawbacks. Therefore, this paper used a method based on deep transfer learning to establish a fitness database. After that, a deep neural network was trained to detect the type and completeness of fitness movements. We used Yolov4 and Mediapipe to instantly detect fitness movements and stored the 1D fitness signal of movement to build a database. Finally, MLP was used to classify the 1D signal waveform of fitness. In the performance of the classification of fitness movement types, the mAP was 99.71%, accuracy was 98.56%, precision was 97.9%, recall was 98.56%, and the F1-score was 98.23%, which is quite a high performance. In the performance of fitness movement completeness classification, accuracy was 92.84%, precision was 92.85, recall was 92.84%, and the F1-score was 92.83%. The average FPS in detection was 17.5. Experimental results show that our method achieves higher accuracy compared to other methods.

## 1. Introduction

Fitness can bring many benefits to the body. With the rise in health awareness, men, women, and children have gradually begun to engage in fitness activities. There are many benefits of fitness exercise; it can effectively improve cardiopulmonary capacity, increase concentration, maintain weight, etc. [1]. Most of those of exercise hope that their posture can be improved, and improving posture can effectively reduce the risk of obesity [2]. Obese bodies are prone to many chronic diseases [3], and each is more likely to lead to death, so regular exercise is important [4].

With the prevalence of COVID-19, people spend less time outdoors [5], which reduces the amount of people’s physical activity. The gym industry, in particular, has been considerably affected, resulting in people being unable to go to the gym to exercise. These athletes then turn to home fitness [6], which can effectively help them avoid contact with people and effectively reduce the impact of the epidemic. In addition, home fitness does not require large fitness equipment but completes fitness exercises through dumbbells, yoga mats, horizontal bars, and other equipment, so it is deeply loved by people. However, people who build their bodies at home usually do not hire fitness trainers but learn fitness-related information from social media and mobile apps. Generally, most athlete are novices and have not received professional fitness exercise guidance, so there is a risk of injury when exercising. Common fitness injuries are usually caused by incorrect posture, heavy equipment, and excessive speed [7]. This type of sports injury is not easy to avoid by obtaining fitness knowledge only through social media. Therefore, a cheap, simple, and accurate fitness movement recognition system is important, which can effectively and instantly detect fitness movements, reduce sports injuries, and improve people’s fitness awareness.

Among them, some systems use wearable devices to detect changes in human body temperature and movement, which, in addition to detecting fitness movements, can also perform preliminary detection of symptoms, such as COVID-19 [8,9]. This method lets the fitness user put on the electronic device, and calculates the three-axis changes of the electronic device when the fitness user is exercising. Then, these data are collected and analyzed using machine learning to classify fitness movements. However, this detection method has some shortcomings. When there are many types of fitness movements, it is difficult to achieve accurate detection. When the body used for the fitness movement is different from the part where the electronic device is worn, it is more difficult to identify the current fitness movement. If the electronic device is carried all over the body, the fitness user will be troubled when exercising, and the cost will be relatively high. Another method is to detect fitness movements based on computer vision, which has lower cost and does not hinder the exercise of fitness users through the detection method of computer vision. The method of detecting fitness movements based on computer vision is further divided into methods based on body nodes and methods based on image deep learning. Body-node-based methods detect fitness movements by calculating body nodes, which can be performed using OpenPose, Mediapipe, Simple Baselines, etc. [10,11,12,13]. Using these methods, nodes of the body and fitness movements can be detected through changes in the coordinates of the nodes. In addition to detecting the speed of fitness movements [10], these methods can also classify the current fitness movement type [11] or the error between fitness movements and standard movements [12,13].

However, these methods cannot detect fitness movements from various angles, especially when the user is on the side or the back, which causes detection errors due to the occlusion of nodes. The last type of detection is a method of detecting fitness movements based on deep learning of images. This type of method usually classifies fitness movements. For example, the convolutional neural network (CNN) method for detecting fitness movements [14] can classify the current fitness movements well. Such classification methods do not cause detection errors due to occlusion of body nodes. As long as the training data of the model are sufficient, fitness movements can be detected from various angles. Usually, this method requires more computation time and cannot detect the nodes of the body in detail. The fitness movement is usually a continuous movement, so if the body nodes cannot be detected in a timely and detailed manner, it is difficult to achieve real-time detection of the fitness movement. Therefore, this paper proposes a method that combines You Only Look Once Version 4 (Yolov4) and Mediapipe to detect fitness movements and uses the multilayer perceptron (MLP) to classify fitness states.

In our method, the deep transfer learning concept is used to train Yolov4 and detect fitness movements. Deep transfer learning is a new type of classification model, which has been widely used in many research fields. Due to the high cost of data collection and labeling, constructing large-scale and sophisticated data is difficult. The use of deep transfer learning can solve the problem of insufficient data. In previous studies, deep transfer learning methods have been applied to the detection of fitness movements [15]. This study corrects for human motion, which is prone to inaccurate detection when detecting complex human movements. The method we propose will also improve the problem of misclassification of fitness movements caused by the loss of Mediapipe nodes during complex movements. We searched for professionally trained fitness trainers and untrained fitness users to capture images and used them to build a database of images. This included labeling of accurate user positions and fitness movements, which were then used to train Yolov4. Finally, Yolov4 was used to initially identify the types of fitness movements and then combined with Mediapipe to detect the nodes of the human body in order to achieve instant and high-precision fitness movement detection and realize completeness of those fitness movements.

## 2. Methods

### 2.1. Proposed System Architecture

To be able to detect the fitness status of various backgrounds, users, shooting angles, and lighting, a sufficient image database is necessary. It takes a considerable amount of time to collect data, and the image data need to go through a long labeling process. This paper proposes a method based on deep transfer learning [16] to detect fitness movements in time and analyze the fitness status.

**Figure 1:**
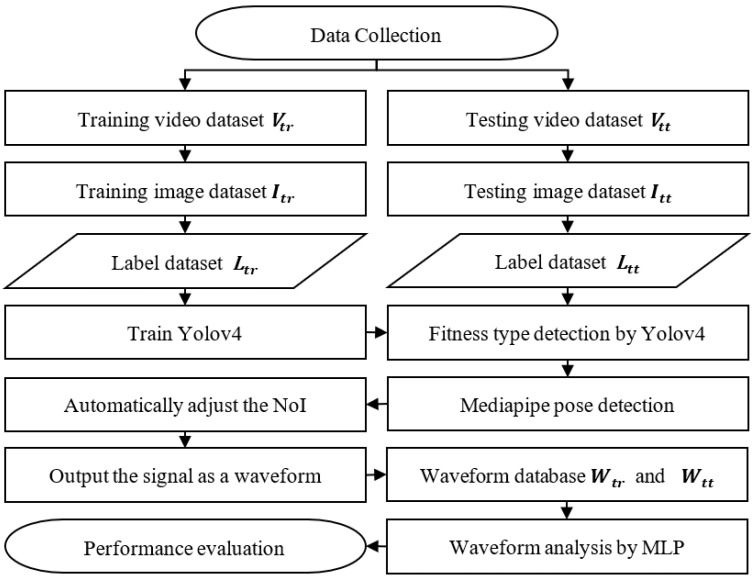
Method flowchart.

First, we collected a sufficient amount of fitness image data, established image database I, and trained Yolov4. We used Yolov4 to judge 12 types of fitness. Afterward, Mediapipe was used to detect the body nodes of fitness users, in which different fitness movements had different nodes of interest (NoI). The current NoI was adjusted based on the detection results of Yolov4. By calculating the angle of the NoI, one can calculate the bending angle of the current joint. The angles of these NoIs were stored as waveforms, and a waveform database W was created. The waveform was then classified by the MLP to detect the fitness status. Finally, the classification performance of Yolov4 and the MLP was evaluated. The flowchart of the proposed method is shown in Figure 1, and the description of the process is as follows:

Step1. Collect 12 types of fitness videos from 20 users and build a video database *V*.
Step2. Divide the video database V into a training set $V_{tr}$ and a test set $V_{tt}$.
Step3. Save in $V_{tr}$ and $V_{tt}$ an image every 10 frames and create image databases $I_{tr}$ and $I_{tt}$.
Step4. Mark the databases ${\;I}_{tr}$ and $I_{tt}$ according to the format of Yolov4 and obtain the databases $L_{tr}$ and $L_{tt}$.
Step5. Use $L_{tr}$ to train Yolov4, obtain the trained weights $W_{f}$, and then use $L_{tt}$ to test the performance of Yolov4.
Step6. Use $V_{tt}$ to detect the fitness type using Yolov4 and the body node of the fitness user using Mediapipe.
Step7. Calculate the angle of the NoI for each fitness movement to obtain the angle of joint flexion.
Step8. According to the fitness type detected by Yolov4, automatically adjust the position of the NoI.
Step9. Output and store the angle calculated by the NoI as a waveform.
Step10. Calculate $Completion_{NoI}$ according to the included angle of the NoI and output it as a 1D waveform.
Step11. Create a database W of the output waveforms and divide them into training set $W_{tr}$ and test set $W_{tt}$.
Step12. Use $W_{tr}$ to train the MLP and $W_{tt}$ to test the MLP’s performance.
Step13. Evaluate the classification performance of Yolov4 and the MLP.

In Figure 1, $V_{tr}$, $I_{tr}$, $L_{tr}$, and $W_{tr}$ represent the data of the training set and $V_{tt}$, $I_{tt}$, $L_{tt}$, and $W_{tt}$ represent the data of the test set. *V* is the video database, *I* is the image database, *L* is the label image database, and *W* is the waveform database.

### 2.2. Dataset for Fitness Types Detection

Sufficient image data can be used to better train deep learning models. For the deep transfer learning method in this paper, a sufficient image database was important. We collected image database I containing whole-body fitness movements, a total of 12 types of fitness movements. The names and images of the movements are shown in Table 1.

#### 2.2.1. Video Dataset

These fitness movements include common fitness movements. In particular, these movements are closer to home fitness. Home fitness is usually performed with simple equipment, such as yoga mats, dumbbells, and horizontal bars, without the need for large fitness equipment. Usually, large fitness equipment has a fixed movement trajectory, but these 12 types of fitness movements are all irregular movement trajectories. That is, different exercise users complete these fitness movements in different postures, which increases the difficulty of image recognition. Therefore, it is difficult to build an image database that can identify these fitness movements. Additionally, these image data go through a labeling process, which is time-consuming and labor-intensive.

In the experiment, 20 users were selected and videos of the 20 users when exercising were used to create a video database *V*. These videos contain 12 types of fitness movements by the 20 users. In the experiment, the users were asked to perform these 12 types of exercises in a row, and each exercise was repeated 3 to 5 times. Every time a fitness movement was performed, the user was required to complete a complete motion trajectory and constantly change the shooting angle. The video format was 30 frames per second, and the length and width were 540 × 540 pixels. Table 2 shows the video time captured when 20 users performed fitness movements. The total shooting time was 62 min and 47 s.

In the selection of fitness exercises, we selected 12 fitness exercises under the advice of fitness trainers, which included chest, back, legs, abs, biceps, triceps, and preparations. These movements can be done using dumbbells or with bare hands, and they are also relatively introductory and popular of all fitness movements. To build the database, professionally trained fitness trainers and users were used to assist in the shooting. After screening, 10 voluntary users were finally selected. The movements of these 10 users were quite standard, so the captured images were used for the training set. Afterward, for fairness in the experiment, another 10 untrained users were found to assist in filming and used the test set data. Since most of the users are not professionally trained, it was better and fairer to use these 10 users for the test set. When the 20 users were shooting images, we instructed them to complete 12 fitness movements, each of which was performed 3 to 5 times according to each user’s habits, with no rest time in between, and completed in the same background.

#### 2.2.2. Image Dataset

The method proposed in this paper was based on deep transfer learning, so Yolov4 training was important. Yolov4 needs to use images for training, so the images in *V* needed to be converted to images. In the *V* video, each fitness movement was completed in 1 to 3 s on average [17]. To record the fitness track from 0% to 100%, the experiment stored the video as an image every 10 frames. Using this method to convert video to image can successfully record the entire fitness track, as shown in Table 3. After training Yolov4 with these images, each user’s continuous movements while exercising are successfully detected. In addition to the complete recording of the motion trajectories, database *I* also contained images of various shooting angles of each fitness movement, as shown in Table 4. The images in database *I* contained complete fitness movement trajectories and images from various angles, which could better train Yolov4. We obtained a total of 13,160 fitness images from 20 users.

Database I contained fitness images of 20 users. To add more users, backgrounds, and shooting angles, this paper collected fitness images online. These images contained screenshots taken from fitness images and videos on platforms such as Youtube and Google. These online images were stored in database I with a pixel size of 540 × 540. The total number of online images was 2964, plus 13,160 fitness images from 20 users. Therefore, database I contained a total of 16,124 images and 12 fitness types.

#### 2.2.3. Image Label

**Figure 2:**
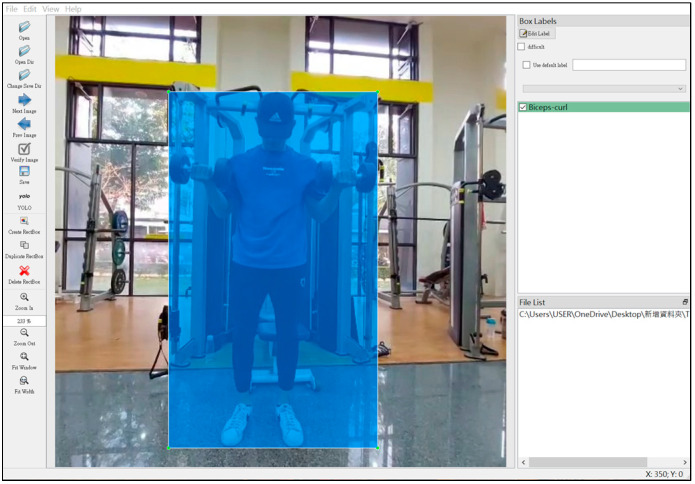
Labeling process using LabelImg.

When database I was prepared, the images were labeled. This paper used the image labeling tool “LabelImg” and performed labeling according to the format required by Yolov4 training. The marking process is shown in Figure 2. The labeling process generates a txt file for each image, which contains the image category and the coordinate position of the object. The markers in the experiment included fitness users, objects on their bodies, and dumbbells.

#### 2.2.4. Training and Testing Dataset Formation

This paper collected a complete image database to train Yolov4 and implemented deep transfer learning so that Yolov4 could better detect fitness movements. To fairly verify the performance of deep transfer learning, the experiments were divided into training and test sets. The training set $V_{tr}$ contained the fitness videos of 10 users, from no. 1 to no. 10 in Table 2. The test set was $V_{tt}$, which contained the fitness videos of 10 users from no. 11 to no. 20. The video in $V_{tr}$ had a longer shooting time because it contained more fitness shooting angles, which enabled Yolov4 to detect more fitness shooting angles. The video in $V_{tt}$ contained only one fitness camera angle and was used to test performance. After that, the videos in video database V were stored every 10 frames, and database I was established. The images in the training set $I_{tr}$ were from $V_{tr}$ and online, while the images in the test set $I_{tt}$ were from $V_{tt}$. Finally, the image database I was labeled to generate training sets $L_{tr}$ and $L_{tt}$, and the number of images is shown in Table 5. $L_{tr}$ contained a total of 12,301 images for training Yolov4, and $L_{tt}$ contained a total of 3823 images for testing the performance of Yolov4.

### 2.3. Dataset for Fitness Completeness Detection

#### 2.3.1. Dataset Preparation

##### Body Nodes Detection

Mediapipe is an open source tool published by Google in 2019. This tool is used for image vision detection. Mediapipe supports many image-vision-based human detection methods, such as face recognition, human body recognition, and gesture recognition [18]. Because Mediapipe supports a variety of programming languages, as well as open source databases, and has high accuracy and fast computing speed, it has been widely used.

**Figure 3:**
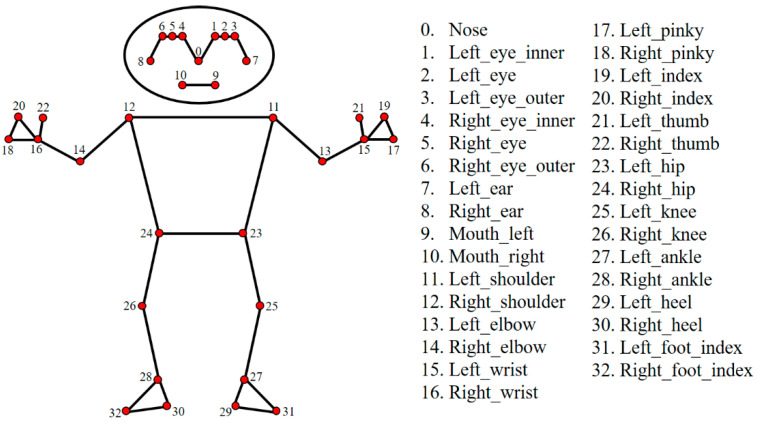
Mediapipe detects 33 nodes of the human pose.

This paper used the Mediapipe BlazePose algorithm provided by Mediapipe, which is a human body detection method that can calculate the 33 nodes of the human body [19], as shown in Figure 3. The algorithm is mainly aimed at the detection of human body posture and can calculate the coordinate position of each joint of the human body. There are 33 such coordinates, ranging from 0 to 32. Except for coordinate 0, “nose,” all other coordinates are symmetrical. Fitness movements are carried out mainly through the movement of the joints of the body, so it is quite suitable to use Medipipe to detect the nodes of joint movements of the body. Human body detection by this method has already been trained, so no additional data collection was required to train the model. Medipipe is great for detecting fitness movements.

**Figure 4:**
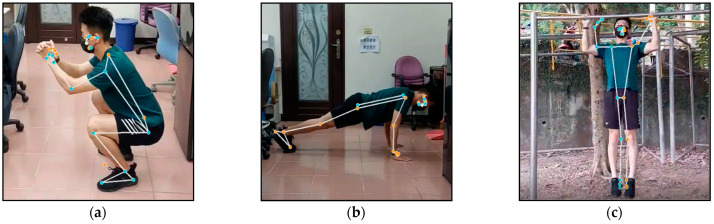
Node missing on Mediapipe detection, (**a**) squat missing node, (**b**) push-up missing node, and (**c**) pull-up missing node.

Currently, there is a method of using Mediapipe to identify fitness movements. This method first uses Mediapipe to identify the nodes of the whole body and obtain the coordinate positions of the nodes. Each coordinate is then used to detect the current fitness category using a K-nearest-neighbor (K-NN) classifier [20]. Using this method, it is simple to count the nodes of the body and detect the fitness category. However, when performing fitness movements, many joints of the body are blocked, which leads to the loss of body nodes detected by Mediapipe. As shown in Figure 4a,b, when exercising with the shooting angle on the side, only half of the body nodes were detected, and the other body nodes were lost. In Figure 4c, the wrist is blocked by the fitness equipment, leading to detection node error. At this time, the loss and error of the body nodes are likely to cause a misjudgment when using the K-NN algorithm to classify the fitness types. However, Yolov4 can solve this problem. Since Yolov4 is a detection method based on image vision, it does not need to rely on node detection of the body. Therefore, as long as training images are sufficient and include a variety of angles, users, and backgrounds, the classification performance of fitness types can be better.

##### Node Angle Detection

This paper combined two methods, Yolov4 and Mediapipe, to detect fitness movements. Yolov4 detects the fitness type, and body nodes are detected by Mediapipe. The results of the two methods for simultaneously detecting fitness movements are shown in Table 6. When the user performs fitness movements, Yolov4 and Mediapipe detect them. At this time, even if the body is blocked by fitness equipment or a node is lost due to the side shooting angle, the detection of the fitness category is not affected. After Yolov4 and Mediapipe detected movements, the key nodes of each fitness movement, that is, NoI, was calculated. The NoI of each fitness movement is shown in Table 6, where the pink node is the NoI of each movement. The angle of the NoI can be calculated, and the completion degree of the current fitness movement can be determined. Through the coordinate positions of the two yellow nodes $P_{1}$ and $P_{3}$ and the NoI node $P_{2}$ in Table 6, the included angle of the NoI can be calculated, and the calculation formula is as follows:

(1) $$Angle_{NoI}=\cos(P_{2})=\frac{P_{2}P_{1}\times P_{2}P_{3}}{\left|P_{2}P_{1}\right|\times |P_{2}P_{3}|}$$

Here $P_{2}P_{1}$ is the vector of $P_{2}$ to $P_{1}$, and $P_{2}P_{3}$ is the vector of $P_{2}$ to $P_{3}$. Through this method, the angle of NoI can be calculated $Angle_{NoI}$, and the fitness completion degree of the current user can be known according to $Angle_{NoI}$.

The NoI is automatically adjusted according to the type of fitness detected by Yolov4. As shown in Table 6, when the user did a squat, the NoI was adjusted to the position of the knee. When the user did a biceps-curl, the NoI was adjusted to the position of the elbow. Therefore, the NoI of each exercise is different from the $Angle_{NoI}$ required to complete the exercise. The position of $P_{1}$, $P_{2}$, and $P_{3}$ and the angle range of $Angle_{NoI}$ for each fitness movement are shown in Table 7 [21]. $Start\_Angle_{NoI}$ indicates the initial angle of the joint when the exercise is ready, and $End\_Angle_{NoI}$ indicates the final bending angle of the joint when the exercise is completed. Among them, “standing” is the preparation movement, so when the user’s movement is “standing,” the NoI does not change and adjust. $Angle_{NoI}$ was adjusted according to $Angle_{NoI}$ calculated by the user in $V_{tr}$ when exercising. The NoI was automatically adjusted by the fitness type detected by Yolov4, and $Angle_{NoI}$ was calculated according to the angle of the NoI. Finally, the fitness completion of the current user was determined through $Angle_{NoI}$. Using this method, the current fitness movement can be detected instantly and accurately.

After Yolov4 and Mediapipe detected the fitness exercise, the user’s fitness type, body joint nodes, and NoI can be obtained. The included angle of NoI can be calculated by $Angle_{NoI}$, and then through the change in $Angle_{NoI}$, one can understand the speed and completion of the user’s fitness. The fitness completion degree was calculated according to $Start\_Angle_{NoI}$ and $End\_Angle_{NoI}$ in Table 7. The fitness completion degree $Completion_{NoI}$ is calculated as follows:

(2) $$Completion_{NoI}=\frac{Angle_{NoI}-Start\_Angle_{NoI}}{End\_Angle_{NoI}-Start\_Angle_{NoI}}$$

Here $Completion_{NoI}$ is between 0 and 1, $Start\_Angle_{NoI}$ indicates the initial angle set by $Angle_{NoI}$ for the fitness movement, and $End\_Angle_{NoI}$ indicates the final angle set by $Angle_{NoI}$ when the fitness movement is completed. $Completion_{NoI}$ indicates the degree of completion of the fitness movement. Generally, completing a complete fitness exercise increases $Completion_{NoI}$ from 0% to 100%, which then decreases to 0%, and this change is stable and slow [22].

#### 2.3.2. Fitness Completeness Definition and Dataset Formation

**Figure 5:**
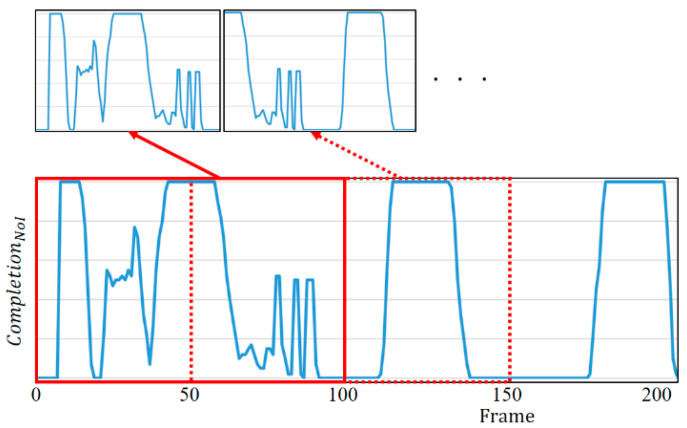
The waveforms of the fitness movements were used for classification.

All videos contained in $V_{tr}$ and $V_{tt}$ were detected by Yolov4 and Mediapipe, and then $Completion_{NoI}$ of the fitness movement was calculated. $Completion_{NoI}$ is displayed in the form of a 1D signal waveform, and database W was created. The 1D wavelines of the video output of $V_{tr}$ and $V_{tt}$ were stored as training set $V_{tr}$ and test set $V_{tt}$. The way of establishing database W is shown in Figure 5, wherein the wave travel of the 1D signal was established by $Completion_{NoI}$ every 100 frames, and the step is 50 frames. As shown in Table 8, after databases $W_{tr}$ and $W_{tt}$ were established, $W_{tr}$ contained a total of 657 records and $W_{tt}$ contained a total of 587 records. These data contained the data of 12 types of fitness movements.

**Figure 6:**
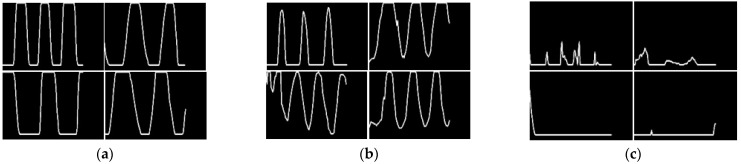
Three categories of waveforms: (**a**) complete, (**b**) no-complete, and (**c**) no-movement.

To perform fitness movements completely, there must be complete range of motion. Therefore, this paper simply divided the 1D waveform data into three categories: complete, no-complete, and no-movement. The three types of waveforms are shown in Figure 6. These categories were judged as follows [21,22]:

- Complete: $Completion_{NoI}$ rose from 0% to 100% and then dropped to 0%, during which the change was stable and slow. In addition, when the value was between 0% and 100%, there was a short stop.
- No-complete: $Completion_{NoI}$ did not rise to 100% or drop to 0% but did not stop at 0% and 100%. In addition, the value change was unstable and fast.
- No-movement: $Completion_{NoI}$ had almost no change, that is, the state of preparation for fitness movements.

### 2.4. Fitness Movement Detection

#### 2.4.1. Fitness Type Detection

Yolo has achieved quite good performance in the task of object detection and also has good performance in detection speed and accuracy [23], so it is widely used in the task of real-time object detection [24]. Fitness moves are continuous, and each move is usually completed in seconds, so a way to detect objects in real time was needed, and Yolo fit the bill.

Yolo continues to improve with this release, with improved object detection accuracy and speed. The Yolov4 method was released in April 2020 [25], and it has received great attention and discussion. Compared with Yolov3, Yolov4 improves 10% AP and 12% frame per second (FPS) and uses the Cross Stage Paritial Darknet 53 (CSPDarknet53) network architecture [26], which can enable Yolov4 to provide faster detection speed and accuracy. In this paper, Darknet was used to train Yolov4. Darknet is an open source neural network architecture [27], which is written in C and CUDA languages, which can train Yolov4 simply and quickly and effectively reduce the training time. Darknet supports the use of the computer’s CPU and GPU for computing, and the use of GPU computing can bring about a faster training speed.

The most important part of the deep transfer learning algorithm proposed in this paper was the training of Yolov4. The complete fitness databases $L_{tr}$ and $L_{tt}$ were used to train and test Yolov4. $L_{tr}$ was added to Darknet and used to train Yolov4 and then obtain weight $W_{f}$. After that, $L_{tt}$ was added to darknet, and $W_{f}$ was used to test the performance of Yolov4. The test results were compared and introduced in later sections.

The video of database $V_{tt}$ was used to test the performance of Yolov4, where the detected results of $V_{tt}$ are shown in Table 9. Each fitness category was successfully detected with a fairly high confidence score. This means that database $L_{tr}$ collected in this paper had enough image data and Yolov4 was fully trained. After the detection of Yolov4, the user’s fitness movement was detected in real time by category and the user’s location.

#### 2.4.2. Fitness Completeness Detection

$W_{tr}$ and $W_{tt}$ contained 1D signals and were divided into three categories. Classifying 1D signals using machine learning methods is a relatively simple task and therefore does not require the use of complex network models. This paper used the MLP to classify these 1D signals [28]. The MLP, also called an artificial neural network (ANN), is a model [29] that belongs to supervised learning. It can quickly solve complex classification problems. The network model contains the input layer of the first layer, the middle hidden layer, and the final output layer. This paper used a 3-layer hidden layer and a 2-layer dense layer and used Dropout to reduce overfitting. The $W_{tr}$ data were used to train the MLP, after which $W_{tt}$ was used to test the performance of the MLP.

## 3. Experimental Section

### 3.1. Experimental Setup

This paper used Yolov4 and Mediapipe to detect fitness movements and finally used the MLP to classify the status of the fitness movements. Among them, Yolov4 and the MLP used the databases $L_{tr}$ and $W_{tr}$ established in this paper for training. Yolov4 was trained using the yolov4.conv.137 network framework in the Darknet network, which includes many experimental settings. The experimental settings for training Yolov4 are shown in Table 10. $L_{tr}$ was used to train Yolov4, and weights $W_{f}$ were obtained after training. The iterations were set to 100,000, but the iterations required for different data types, the number of categories, and data quantities were different. To understand the iterations required for database $L_{tr}$ built in this paper, the iterations were 10,000, 20,000…100,000 to train Yolov4 and compare its performance. The experimental settings for training the MLP are shown in Table 11, using $W_{tr}$ to train the MLP. Since the data size in $W_{tr}$ was a 1D signal of 100, it is not like Yolov4 had length and width and image channels, but the data size was set to 100.

### 3.2. Evaluation Index

When Yolov4 and the MLP were trained, performance was tested. Among them, Yolov4 obtained 12 types of detection results and the MLP obtained 3 types of detection results, both of which belonged to the classification methods in machine learning. According to the classification results of each category, true positives (*TPs*), false positives (*FPs*), false negatives (*FNs*), and true negatives (*TNs*) were obtained. The introduction of these four evaluation indicators is as follows:

- *TP*: positive samples predicted by the model to be positive
- *FP*: negative samples predicted by the model to be positive classes
- *FN*: positive samples predicted by the model to be negative
- *TN*: negative samples predicted by the model to be negative classes

According to the number of *TPs*, *FPs*, *FNs*, and *TNs*, the classification performance of Yolov4 and the MLP can be understood. When the number of *TPs* is large, it indicates that the number of correct classifications for the experiment is greater. Then, *accuracy*, *precision*, *recall*, and the *F1-score* were calculated as follows:

(3) $$Accuracy=\frac{TP+TN}{TP+TN+FP+FN}$$

(4) $$Precision=\frac{TP}{TP+FP}$$

(5) $$Recall=\frac{TP}{TP+FN}$$

(6) $$F1\;score=2\times \frac{Precision\times Recall}{Precision+Recall}$$

In addition, the predicted box detected in Yolov4 was evaluated using the intersection over union (*IoU*). The calculation method of the *IoU* is as follows:

(7) $$IoU=\frac{Area\;of\;Overlap}{Area\;of\;Union}$$

Here, *area of overlap* represents the area where the actual box overlaps with the estimated box. The *area of union* represents the area of the union of the actual box and the estimated box. The larger the estimated overlap area with the actual box, the better the performance.

In addition to these performance evaluation indicators, the mean average precision (mAP), FPS, and Yolov4 training time were also used as evaluation indicators. Among them, there are many methods of evaluating the mAP. This paper used the PascalVOC 2010–2012 mAP algorithm [30]. The FPS is the number of frames per second that can be calculated when Yolov4 detects the video of $L_{tt}$. Finally, the training time of Yolov4 was also evaluated, and the training time was affected by the iteration. Therefore, later sections will evaluate the changes in detection performance for different iterations and compare them.

### 3.3. Results and Discussion

**Figure 7:**
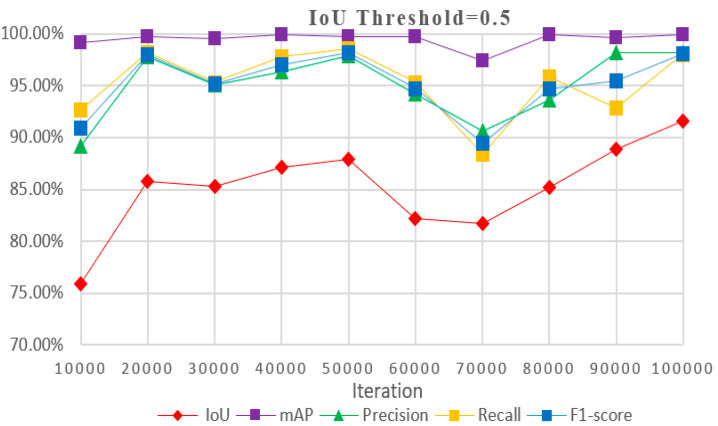
When the IoU threshold was 0.5, the performance comparison of different iterations.

**Figure 8:**
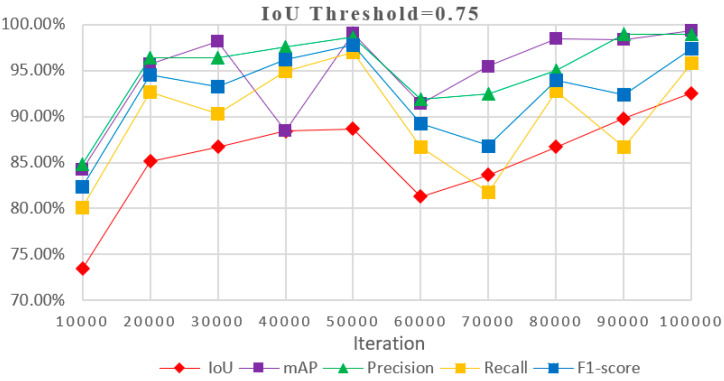
When the IoU threshold was 0.75, the performance comparison of different iterations.

The performance of Yolov4 is shown first, but before entering the performance evaluation, the iteration settings of Yolov4 training were compared and the iterations were 10 settings to train Yolov4: 10,000, 20,000...100,000. Although the higher the iteration setting is, the loss will generally continue to decrease, but this also increases the time cost of training and there is a risk of overfitting. So, a suitable iteration should be found and trained. $L_{tr}$ was used to train Yolov4, and $L_{tt}$ was used to test the performance of Yolov4. The performance of Yolov4 at different iterations is shown in Figure 7 and Figure 8. In the performance comparison, the IoU thresholds were set at 0.5 and 0.75. The experimental results showed that when the iteration was set to 50,000, high performance was obtained. After the iteration exceeded 50,000, the performance decreased, and the performance did not improve until the iteration was 100,000. So considering the time cost of training, the iterations were set to 50,000.

There were two performances in the experimental results. The first was the performance of using Yolov4 to detect fitness movement categories and the second the performance of using the MLP to classify fitness movement 1D signal waveforms. $L_{tt}$ was used to test the performance of Yolov4, and $W_{tt}$ was used to test the performance of the MLP. The experimental results of Yolov4 are shown in Table 12. The experimental results showed that mAP achieved high performance. This is because the image in $L_{tt}$ contained only one fitness user, that is, only one fitness user appears in each image. However, the mAP showed high performance, which means that database $L_{tr}$ completely trained Yolov4. The results showed that when the IoU threshold was set to 0.5, the accuracy was 98.56%, precision was 97.9%, recall was 98.56%, and the F1-score was 98.23%. The FPS averaged 17.5 when running on a laptop with an i7-1185G7 CPU and a GTX-1650Ti GPU. This means that when there are 12 fitness movements in the category, Yolov4’s fitness category detection achieves quite high performance and has the ability to process in real time. To avoid detection errors of several frames, in the detection of $V_{tt}$, a buffer of 15 frames was set, which is equivalent to a buffer time of 0.5 s. Only when 15 frames of images are incorrectly detected will the current fitness type detected by Yolov4 change and the position of NoI will change. That is, according to the experimental results in Table 12, when $V_{tt}$ is detected by Yolov4, it is difficult for the fitness type to be detected incorrectly.

**Figure 9:**
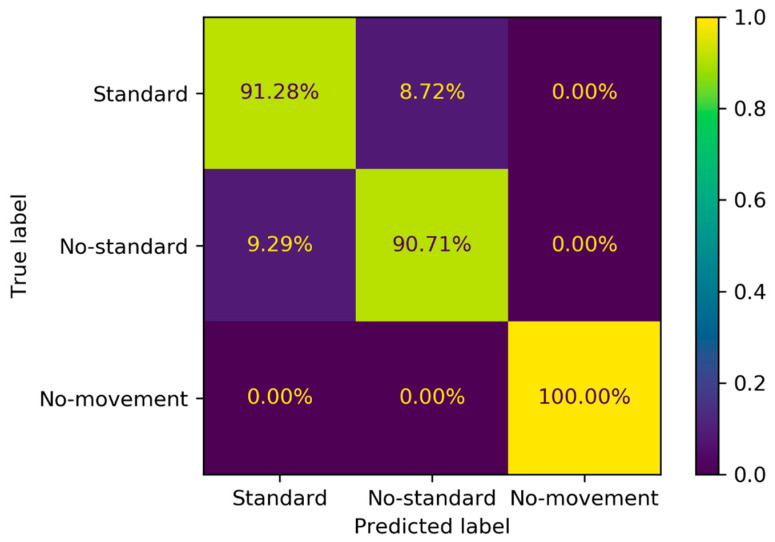
Confusion matrix for MLP classification performance.

The results of classifying $W_{tt}$ using the MLP are shown in Table 13. The results showed that the accuracy was 92.84%, precision was 92.85, recall was 92.84%, and the F1-score was 92.83%. Although this classification result did not achieve high performance, it could still effectively classify the fitness status. The confusion matrix of the MLP classification results is shown in Figure 9, which shows that the classification results of complete and no-complete are poor. This is because the videos in database *V* were not specifically required to perform the complete and no-complete fitness movements when the videos were shot. Therefore, there is not a great difference between these two categories. Although a small amount of $W_{tt}$ data is classified into different categories, the wave patterns are similar. Although the classification performance of the MLP is degraded due to this factor, it still provides valid classification results.

In this paper, a method based on deep transfer learning was used to build a complete database to train and test Yolov4. Yolov4 is an image detection method based on deep learning. This paper used Yolov4 to classify fitness movements.

The methods previously introduced by Hobeom Jeon et al. [11] and Ali Bidaran et al. [14] are both image-based motion detection methods, and both are classified by machine learning. The method of Yongpan Zou et al. [31] and Crema et al. [32] is to let the user wear an electronic wearable device to classify fitness movements through the signals of the electronic device. These methods all classify fitness movements, so the method proposed in this paper was compared with these methods. The experimental results are shown in Table 14 and Table 15. Our method had an mAP of 99.71% and an accuracy of 98.56%. Compared to Hobeom Jeon et al. [11], the mAP improved performance by 9.21%. Compared to Yongpan Zou et al. [31], Crema et al. [32], and Ali Bidaran et al. [14], accuracy improved the performance by 2.49%, 4.2%, and 5.66%, respectively. This result shows that our deep-transfer-learning-based method can provide better classification performance and lead to better detection results for subsequent fitness movements.

In the analysis of fitness movements, we divided the completion of fitness movements into three categories and use the MLP to classify them. The experimental results are shown in Table 16; the accuracy of our method was 92.84%. Compared to the method of Yongpan Zou et al. [31], accuracy improved the performance by 2.14%. Our method is cheaper and does not have to consider the power consumption and hygiene issues of wearable devices. Compared to the method of Jiangkun Zhou et al. [12], accuracy improved the performance by 29.65%. Experimental results showed that our proposed method has better performance.

In the methods of Madanayake et al. [33] and Chen et al. [34], the Kinect sensor was used to analyze fitness movements. Compared to our method, it increases image depth and also increases the cost. This method can successfully detect fitness movements, but the experimental results have not shown its performance, so it cannot be compared.

According to the experimental results and the performance comparison with other methods, the method proposed in this paper has the following contributions:

- This paper proposed a low-cost and effective method for current research on image-based fitness motion detection. This method has the advantages of low cost and real-time processing, and images captured by ordinary smartphones and network cameras can be used to detect fitness movements. It is proved by the experimental results that the method proposed in this paper can be practically applied to a variety of different users, and the detection performance is effective and immediate.
- The method proposed in this paper does not require a professionally trained fitness trainer but trains Yolov4 and detects fitness movements through deep transfer learning. To achieve high-precision detection and fair performance evaluation, this paper collected images of 20 users and online images for training and testing Yolov4. The experimental results show that the database collected in this paper is sufficient to train Yolov4, and it can detect fitness movements under different angles, backgrounds, and users’ shots.
- This paper proposed a method combining Yolov4 and Mediapipe to detect fitness movements. Using Yolov4 to detect fitness categories can reduce errors caused by missing nodes and can detect fitness types from more angles. By further using Mediapipe to detect body nodes, one can understand the movement changes in the body in more detail and automatically adjust the position of the NoI according to the fitness type detected by Yolov4, which can effectively reduce the misjudgment of invalid nodes of the body and focus on valid nodes.
- This paper proposed a method of using the MLP to detect 1D signal waveforms of fitness movements. This must rely on a method to automatically adjust the NoI, calculate the angle of the NoI, and detect the fitness completion and speed of the fitness user. Using this method, the current state of fitness can be classified simply and effectively and the basic fitness state classification results of fitness users can be obtained.

This method can detect fitness movements in real time, but there are still many areas that can be improved, which can be considered in the future as follows:

- The deep learning methods used in this paper include Yolov4, Mediapipe, and the MLP. Therefore, in the future, adding some other machine learning algorithms, such as Genetic Algorithm, can used greatly improve performance [35].
- In this paper, 20 users were selected to assist in shooting fitness images, and an image database was established. However, these images required a lot of labor when marking them. In addition, when shooting these images, the background is usually the same. Therefore, in the future, we will consider using image processing to automatically identify fitness users and automatically mark them. This can greatly reduce personnel use and effectively increase the number of images.
- In this study, 20 users and 12 fitness movements were used for training. Another 10 users were used for testing our system. In the future, we will increase the number of users and the number of fitness movements.

## 4. Conclusions

This paper proposed a method for detecting fitness movements based on deep transfer learning, which is an image-based method and has the advantages of low cost, timeliness, and accuracy. The method is mainly divided into four stages to complete, namely image database collection, Yolov4 detection of fitness categories, Mediapipe detection of body nodes and joint angles, and MLP classification of fitness 1D signal waveforms. This paper collected 20 users and online image data to train Yolov4 and detect the type of fitness movements. After that, Yolov4 and Mediapipe were combined to further detect the nodes of the body, which were used to calculate the joint angle of the body NoI during fitness. Finally, the change in angle was converted into a 1D fitness signal waveform, and the MLP was used to classify it. The experimental results showed that Yolov4, which is based on deep transfer learning training, has good classification performance for the detection of fitness movements. Among them, the mAP was 99.71%, accuracy was 98.56%, precision was 97.9%, recall was 98.56%, the F1-score was 98.23%, and the average FPS was 17.5, which means its classification performance is timely and accurate. This means that the image database collected in this paper can fully train Yolov4, which can produce good classification results for subsequent research on fitness detection. In the experiment of MLP classification of fitness 1D signal waveforms, the accuracy was 92.84%, precision was 92.85%, recall was 92.84%, and the F1-score is was 92.83%. This classified the 1D signal waveforms of fitness movements and obtained valid results. Compared to other methods, our proposed method has better performance. The experimental results show that the method proposed in this paper can effectively, timely, and accurately classify fitness movements and can effectively detect the current fitness state.

## Figures and Tables

**Table 1:** Twelve types of fitness movements and names.

Squat	Push-Up	Pull-Up	Sit-Up
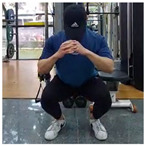	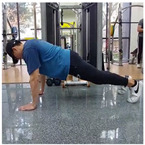	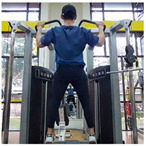	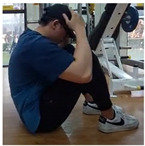
Standing	Biceps-curl	Bulgarian-squat	Bench-press
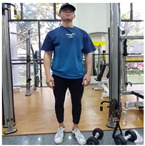	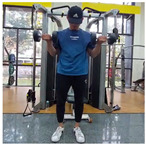	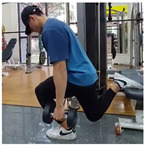	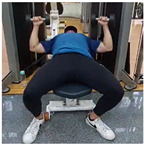
Lateral-raise	Overhead-press	Dumbbell-rowing	Triceps-extension
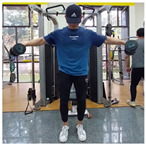	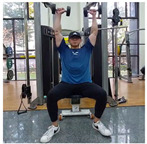	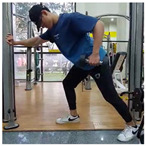	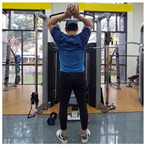

**Table 2:** The fitness time photographed by each user.

User (No.)	Time (s)	User (No.)	Time (s)
1	228	11	169
2	246	12	136
3	246	13	168
4	172	14	193
5	190	15	157
6	177	16	146
7	191	17	170
8	300	18	191
9	170	19	192
10	168	20	157

**Table 3:** The motion track recorded after converting the video database to images.

0%	25%	50%	75%	100%
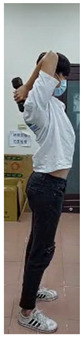	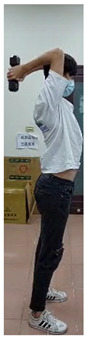	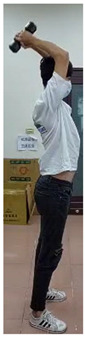	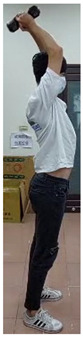	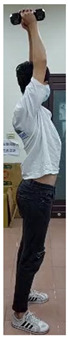

**Table 4:** In the image database, the shooting angle included in each fitness exercise.

0°	45°	90°	135°	180°
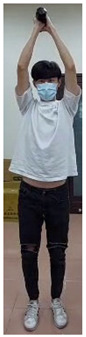	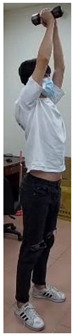	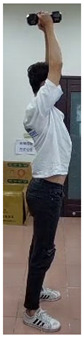	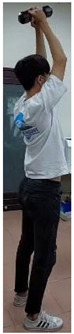	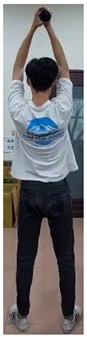

**Table 5:** Category and number of images.

Fitness	$\;L_{tr}\;(\mathrm{Online})$	$\;L_{tr}$	$\;L_{tt}$
Squat	198	644	121
Pull-up	239	1442	427
Push-up	317	913	264
Sit-up	373	977	328
Standing	132	1065	454
Biceps-curl	273	405	154
Bulgarian-split-squat	311	577	365
Bench-press	304	924	471
Lateral-raise	162	299	152
Overhead-press	202	724	365
Dumbbell-rowing	305	598	347
Triceps-extension	148	769	384
Total	2964	9337	3823

**Table 6:** Result of Mediapipe and Yolov4 detecting fitness.

Squat	Push-Up	Pull-Up	Sit-Up
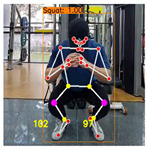	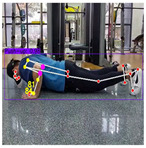	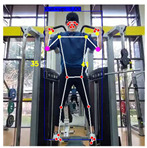	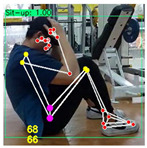
Standing	Biceps-curl	Bulgarian-squat	Bench-press
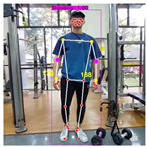	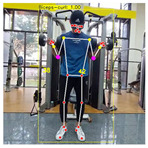	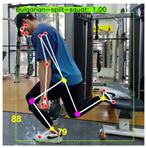	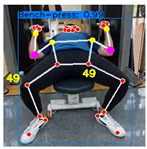
Lateral-raise	Overhead-press	Dumbbell-rowing	Triceps-extension
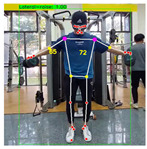	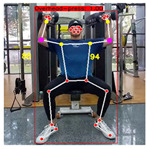	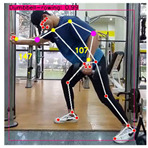	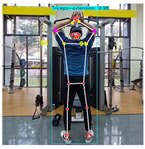

**Table 7:** The position of $P_{1}$, $P_{2}$, and $P_{3}$ corresponding to the body node in Figure 3.

Fitness	$P_{1}$	$P_{2}(\mathrm{NoI})$	$P_{3}$	$\mathrm{Start}\_{\mathrm{Angle}}_{\mathrm{NoI}}$	$\mathrm{End}\_{\mathrm{Angle}}_{\mathrm{NoI}}$
Squat	24	26	28	100	170
Pull-up	12	14	16	80	170
Push-up	12	14	16	80	170
Sit-up	12	24	26	100	120
Standing	×	×	×	×	×
Biceps-curl	12	14	16	80	160
Bulgarian-split-squat	24	26	28	110	160
Bench-press	12	14	16	80	140
Lateral-raise	14	12	24	20	80
Overhead-press	12	14	16	80	150
Dumbbell-rowing	12	14	16	110	150
Triceps-extension	12	14	16	80	140

**Table 8:** The distribution of training and testing data in database W.

	$W_{\mathrm{tr}}$	$W_{\mathrm{tt}}$
Complete	223	203
No-complete	372	261
No-movement	62	123
Total	657	587

**Table 9:** Result of Yolov4 detecting fitness type.

Squat	Push-Up	Pull-Up	Sit-Up
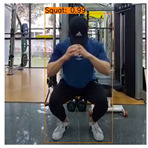	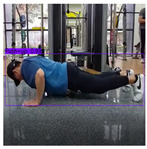	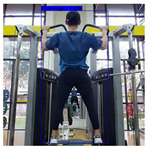	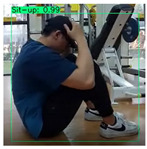
Standing	Biceps-curl	Bulgarian-squat	Bench-press
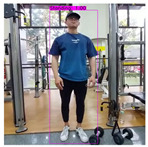	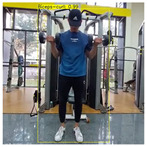	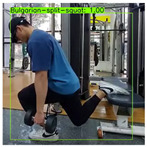	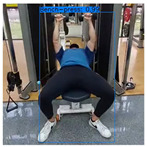
Lateral-raise	Overhead-press	Dumbbell-rowing	Triceps-extension
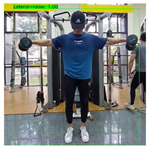	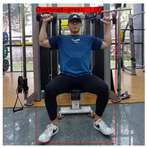	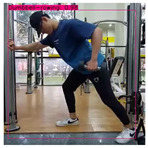	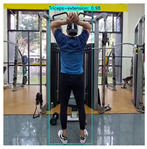

**Table 10:** Experimental setup for training Yolov4.

Parameters	Value
Class	12
Batch size	64
Subdivisions	40
Image width	416
Image height	416
Channels	3
Max batches	100,000
Filters	51
Learning rate	0.001
Decay	0.0005

**Table 11:** Experimental setup for training the MLP.

Parameters	Value
Class	3
Batch size	64
Data size	100
Max batches	100
Learning rate	0.001
Decay	0.0001

**Table 12:** Performance of Yolov4.

Evaluation Index	IoU Threshold = 0.5	IoU Threshold = 0.75
mAP	99.71%	99.08%
Accuracy	98.56%	96.97%
Precision	97.90%	98.67%
Recall	98.56%	96.97%
F1-score	98.23%	97.82%

**Table 13:** Performance of the MLP.

Evaluation Index	MLP
Accuracy	92.84%
Precision	92.85%
Recall	92.84%
F1-score	92.83%

**Table 14:** Comparison of the mAP for fitness movement classification.

Evaluation Index	mAP
Ours	99.71%
Hobeom Jeon et al. [11]	90.5%

**Table 15:** Comparison of accuracy of fitness movement classification.

Evaluation Index	Accuracy
Ours	98.56%
Yongpan Zou et al. [31]	96.07%
Crema et al. [32]	94.36%
Ali Bidaran et al. [14]	92.9%

**Table 16:** Comparison of accuracy of fitness movement analysis.

Evaluation Index	Accuracy
Ours	92.84%
Yongpan Zou et al. [31]	90.7%
Jiangkun Zhou et al. [12]	59.7%

## Data Availability

Not applicable.

## References

[1] Kaminsky L.A., Arena R., Ellingsen Ø., Harber M.P., Myers J., Ozemek C., Ross R. (2019). Cardiorespiratory fitness and cardiovascular disease-the past, present, and future. Prog. Cardiovasc. Dis..

[2] Myers J., Kokkinos P., Nyelin E. (2019). Physical activity, cardiorespiratory fitness, and the metabolic syndrome. Nutrients.

[3] Censin J.C., Peters S.A., Bovijn J., Ferreira T., Pulit S.L., Mägi R., Mahajan A., Holmes M.V., Lindgren C.M. (2019). Causal relationships between obesity and the leading causes of death in women and men. PLoS Genet..

[4] Walter R. (2019). Thompson Worldwide survey of fitness trends for 2019. ACSM’s Health Fit. J..

[5] Nyenhuis S.M., Greiwe J., Zeiger J.S., Nanda A., Cooke A. (2020). Exercise and fitness in the age of social distancing during the COVID-19 pandemic. J. Allergy Clin. Immunol. Pract..

[6] Joo S.Y., Lee C.B., Joo N.Y., Kim C.R. (2021). Feasibility and effectiveness of a motion tracking-based online fitness program for office workers. Healthc. Multidiscip. Digit. Publ. Inst..

[7] Rynecki N.D., Siracuse B.L., Ippolito J.A., Beebe K.S. (2019). Injuries sustained during high intensity interval training: Are modern fitness trends contributing to increased injury rates?. J. Sports Med. Phys. Fit..

[8] Merenda M., Astrologo M., Laurendi D., Romeo V., Della Corte F.G. A Novel Fitness Tracker Using Edge Machine Learning. Proceedings of the 2020 IEEE 20th Mediterranean Electrotechnical Conference (MELECON).

[9] Daskalos A.-C., Theodoropoulos P., Spandonidis C., Vordos N. (2022). Wearable Device for Observation of Physical Activity with the Purpose of Patient Monitoring Due to COVID-19. Signals.

[10] Zhao Z., Lan S., Zhang S. Human Pose Estimation based Speed Detection System for Running on Treadmill. Proceedings of the 2020 International Conference on Culture-oriented Science & Technology (ICCST).

[11] Jeon H., Yoon Y., Kim D. Lightweight 2D human pose estimation for fitness coaching system. Proceedings of the 2021 36th International Technical Conference on Circuits/Systems, Computers and Communications (ITC-CSCC).

[12] Zhou J., Feng W., Lei Q., Liu X., Zhong Q., Wang Y., Jin J., Gui G., Wang W. Skeleton-based Human Keypoints Detection and Movement Similarity Assessment for Fitness Assistance. Proceedings of the 2021 IEEE 6th International Conference on Signal and Image Processing (ICSIP).

[13] Pauzi A.S.B., Mohd Nazri F.B., Sani S., Bataineh A.M., Hisyam M.N., Jaafar M.H., Ab Wahab M.N., Mohamed A.S.A. (2021). Movement Estimation Using Mediapipe BlazePose. Proceedings of the International Visual Informatics Conference.

[14] Bidaran A., Sharifian S. Designing an AI-assisted toolbox for fitness activity recognition based on deep CNN. Proceedings of the 2021 12th International Conference on Information and Knowledge Technology (IKT).

[15] Ke Y., CanNan Z.E.N.G., XingHua L.U., YuHan C.U.I. Recognition technology of human body movement behavior in fitness exercise based on transfer learning. Proceedings of the 2021 6th International Conference on Intelligent Computing and Signal Processing (ICSP).

[16] Tan C., Sun F., Kong T., Zhang W., Yang C., Liu C. (2018). A survey on deep transfer learning. Proceedings of the International Conference on Artificial Neural Networks.

[17] Palmieri G.A. (1987). Weight training and repetition speed. J. Strength Cond. Res..

[18] Lugaresi C., Tang J., Nash H., McClanahan C., Uboweja E., Hays M., Zhang F., Chang C.L., Yong M.G., Lee J. (2019). Mediapipe: A framework for building perception pipelines. arXiv.

[19] Bazarevsky V., Grishchenko I., Raveendran K., Zhu T., Zhang F., Grundmann M. (2020). Blazepose: On-device real-time body pose tracking. arXiv.

[20] Halder A., Tayade A. (2021). Real-time vernacular sign language recognition using mediapipe and machine learning. Int. J. Res. Publ. Rev..

[21] Baechle T.R., Earle R.W. (2019). Weight Training: Steps to Success.

[22] Frost D., Andersen J., Lam T., Finlay T., Darby K., McGill S. (2013). The relationship between general measures of fitness, passive range of motion and whole-body movement quality. Ergonomics.

[23] Redmon J., Divvala S., Girshick R., Farhadi A. You only look once: Unified, real-time object detection. Proceedings of the IEEE conference on computer vision and pattern recognition.

[24] Shinde S., Kothari A., Gupta V. (2018). YOLO based human movement recognition and localization. Procedia Comput. Sci..

[25] Bochkovskiy A., Wang C.Y., Liao H.Y.M. (2020). Yolov4: Optimal speed and accuracy of object detection. arXiv.

[26] Jiang Z., Zhao L., Li S., Jia Y. Real-time object detection method for embedded devices. Proceedings of the Computer Vision and Pattern Recognition.

[27] Wang C.Y., Bochkovskiy A., Liao H.Y.M. Scaled-yolov4: Scaling cross stage partial network. Proceedings of the IEEE/cvf Conference on Computer Vision and Pattern Recognition.

[28] Popescu M.C., Balas V.E., Perescu-Popescu L., Mastorakis N. (2009). Multilayer perceptron and neural networks. WSEAS Transmovements Circuits Syst..

[29] Grossi E., Buscema M. (2007). Introduction to artificial neural networks. Eur. J. Gastroenterol. Hepatol..

[30] Everingham M., Van Gool L., Williams C.K., Winn J., Zisserman A. (2009). The pascal visual object classes (voc) challenge. Int. J. Comput. Vis..

[31] Zou Y., Wang D., Hong S., Ruby R., Zhang D., Wu K. (2022). A low-cost smart glove system for real-time fitness coaching. IEEE Internet Things J..

[32] Crema C., Depari A., Flammini A., Sisinni E., Haslwanter T., Salzmann S. IMU-based solution for automatic detection and classification of exercises in the fitness scenario. Proceedings of the 2017 IEEE Sensors Applications Symposium (SAS).

[33] Madanayake P.S., Wickramasinghe W.A.D.K., Liyanarachchi H.P., Herath H.M.D.M., Karunasena A., Perera T.D. Fitness Mate: Intelligent workout assistant using motion detection. Proceedings of the 2016 IEEE International Conference on Information and Automation for Sustainability (ICIAfS).

[34] Chen C., Liu K., Jafari R., Kehtarnavaz N. Home-based senior fitness test measurement system using collaborative inertial and depth sensors. Proceedings of the 2014 36th Annual International Conference of the IEEE Engineering in Medicine and Biology Society.

[35] Caponetto R., Fortuna L., Graziani S., Xibilia M.G. (1993). Genetic algorithms and applications in system engineering: A survey. Trans. Inst. Meas. Control.

